# Pulmonary function, respiratory symptoms, and dust exposures among workers engaged in early manufacturing processes of tea: a cohort study

**DOI:** 10.1186/1471-2458-12-121

**Published:** 2012-02-13

**Authors:** Tzong-Shiun Shieh, Jui-Jung Chung, Chung-Jing Wang, Perng-Jy Tsai, Yau-Chang Kuo, How-Ran Guo

**Affiliations:** 1 Department of Family Medicine and Occupational Medicine Center, St. Martin De Porres Hospital, Chia-Yi, Taiwan; 2 Department of Environmental and Occupational Health, National Cheng Kung University, Tainan, Taiwan; 3 Medical Administration Department, St. Martin De Porres Hospital, Chia-Yi, Taiwan; 4 Department of Occupational and Environmental Medicine, National Cheng Kung University, Tainan, Taiwan; 5 Department of Occupational and Environmental Medicine, National Cheng Kung University Hospital, Tainan, Taiwan; 6 Sustainable Environment Research Center, National Cheng Kung University, Tainan, Taiwan; 7 Medical Device Innovation Center, National Cheng Kung University, Tainan, Taiwan

**Keywords:** Tea, Occupational exposure, Pulmonary function tests, Signs and symptoms, Respiratory

## Abstract

**Background:**

To evaluate pulmonary function and respiratory symptoms in workers engaged in the early manufacturing processes of tea and to identify the associated factors, we conducted a study in a tea production area in Taiwan.

**Methods:**

We recruited tea workers who engaged in the early manufacturing process in the Mountain Ali area in Taiwan and a comparison group of local office workers who were matched for age, gender, and smoking habits. We performed questionnaire interviews, pulmonary function tests, skin prick tests, and measurement of specific IgE for tea on the participants and assessed tea dust exposures in the tea factories.

**Results:**

The 91 participating tea workers had higher prevalence of respiratory symptoms than the comparison group (32 participants). Among tea workers, ball-rolling workers had the highest prevalence of symptoms and the highest exposures of inhalable dusts. At baseline, tea workers had similar pulmonary functions as the comparison group, but compared to the other tea workers ball-rolling workers had a lower ratio of the 1-second forced expiratory volume to forced vital capacity (FEV_1_/FVC) and a lower maximal mid-expiratory flow rate expressed as% of the predicted value--MMF (%pred). A total of 58 tea workers participated in the on-site investigation and the cross-shift lung function measurements. We found ball-rolling yielded the highest inhalable dust level, panning yielded the highest respirable dust level, and withering yielded the lowest levels of both dusts. Ball-rolling also yielded the highest coarse fraction (defined as inhalable dusts minus respirable dusts), which represented exposures from nose to tracheobronchial tract. During the shift, we observed significant declines in pulmonary function, especially in ball-rolling workers. Multiple regressions showed that age, height, work tasks, coarse fraction, and number of months working in tea manufacturing each year were independent predictors of certain pulmonary function parameters in tea workers.

**Conclusions:**

Tea workers engaged in early manufacturing processes of tea have higher prevalence of respiratory symptoms and pulmonary function impairment, which might be related to tea dust exposures, especially the coarse fraction.

## Background

**Figure 1:**
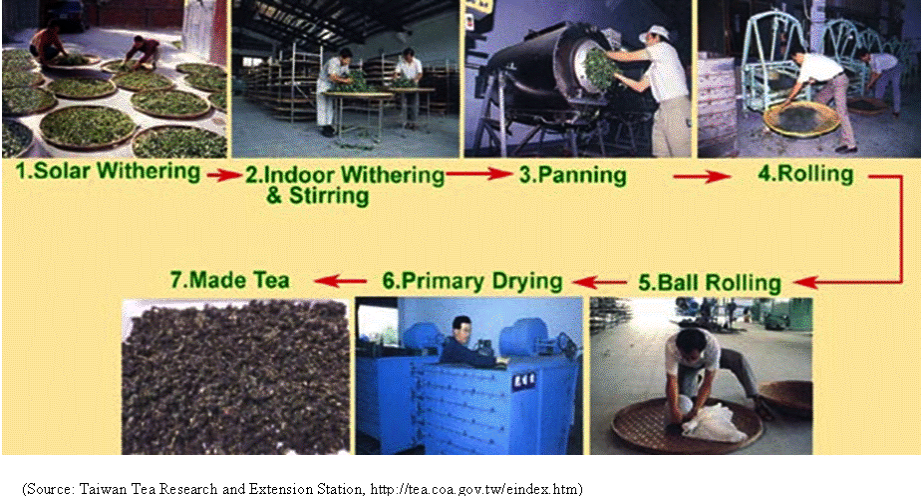
**The manufacturing process of semi-fermented tea**. (Source: Taiwan Tea Research and Extension Station)

Tea is a popular beverage all over the world. Tea is made of the leaves and leaf buds of *Camellia sinensis*, and the manufacturing processes can be divided into two stages. The early stage includes withering, panning, rolling, ball-rolling, and drying (Figure 1), and the late stage includes sifting and blending, packing, and other processes before export and trade. Common teas can be classified into non-fermented tea (green tea, yellow tea, etc.), semi-fermented tea (Oolong tea, Paochong tea, etc.), and fully-fermented tea (black tea), but all are produced through a series of processes. The withering process includes solar and indoor withering, which allows the polyphenol catechins in leaves to be oxidized, a process called "fermentation." This process gives the tea its color and taste. Panning stops the fermentation and evaporates the water in leaves by the high temperature of the panning machine. Rolling rubs and destructs the surface tissues of leaves which results in liquid coating on the surface and makes the tea easy to brew. Ball-rolling involves rotating and pressing the dried leaves in a sack and turns leaves into strip and ball shapes, which requires considerable amount of labors. The final process is drying, in which water evaporates further. The tasks are different from those of the late-stage tea manufacturing, which might have dusts from fungal origins [1].

Occupational asthma has been described among workers involved in packaging and blending [2-4]. Acute symptoms, including cough, chest tightness, and rhinorrhea, were observed by some studies in tea workers during their work, and some studies found higher prevalence of chronic respiratory symptoms among blending workers [5-8]. Exposures to tea dusts could cause airway obstruction [9-11], and higher prevalence of sensitization to tea was found in tea workers, especially herbal tea workers [11,12], although the relations of tea sensitization status to respiratory symptoms and function were unclear [8,11,12]. There are, however, few, if any, studies on early-stage manufacturing.

The two stages of tea manufacturing have differences in work schedule, workplace, processes, and materials, which may lead to variations in the intensity, frequency, and contents of tea dust exposure. Therefore, workers engaged in different stages might suffer from different respiratory effects. We conducted a study to assess respiratory symptoms, pulmonary function, and tea dust exposure of workers engaged in early-stage tea manufacturing and to identify contributing factors of respiratory effects.

## Methods

### Study populations

Tea manufacturing is an important industry in Taiwan, and high-grade semi-fermented teas such as Paochong and Oolong teas are the feature products. There are about 6,000 tea factories and 25,000 tea workers engaged in early-stage tea manufacturing. We conducted a study in the Mountain Ali, a major tea production area in Taiwan.

We selected 95 workers randomly from male workers who were 20 to 60 years old and had worked in tea-manufacturing factories for 1 year or more. For comparison, we recruited 40 male office workers from the local Forestry Bureau office who were 20 to 60 years of age but had never worked in the tea industry. They were frequency-matched for smoking habits because smoking habit is an important factor affecting respiratory symptoms and pulmonary function. The main advantage of using office workers working in the same region for comparison is that the effects of environmental exposures could be minimized. In addition, other blue collar workers generally have more occupational exposures to respiratory hazards than office workers, which may affect the evaluation of the effects of tea dusts. We excluded those who had cardiopulmonary diseases before working for the tea factories or the Bureau and those who had known contacts with respiratory hazards before or during working for the tea factories or the Bureau. This study was approved by the Grant Review Committee of the St. Martin De Porres Hospital.

### Baseline assessments of respiratory symptoms, pulmonary function, and sensitization status

We performed baseline assessments on both groups in August, about 2 weeks before the autumn tea production term. A WHO respiratory symptom questionnaire for early detection of respiratory diseases was modified to fit the local conditions and administered in the study [13]. Accordingly, we defined "chronic cough or phlegm" as cough or phlegm production on most days (> 4 day/week) for at least three months per year, "chronic bronchitis" as cough with phlegm production for a minimum of 3 months a year for at least two consecutive years, "dyspnea" as shortness of breath when walking with other people at an ordinary pace or at their own pace on the ground level, and "wheeze, chest tightness, nasal catarrh, eye discomfort/dryness" as having the symptoms two times or more in the recent year, excluding those with known causes such as common cold and conjunctivitis. A "work-related" symptom indicated the new onset or exacerbation of a chronic symptom during the work shift. Physical examinations were conducted by an experienced physician and included measurements of body height and weight as well as evaluations of cardiovascular and respiratory systems.

Pulmonary function tests were performed using a spirometer (Spiroanalyzer ST-90, Fukuda Sangyo, Tokyo, Japan), which was calibrated before and after each round of measurements. The procedure was carried out by a well-trained technician, who was blinded to the exposure status of participants and performed the tests according to the American Thoracic Society guidelines [14-16]. At least two practice blows were performed, and the highest reading was adopted from at least two readings. We measured the forced vital capacity (FVC), 1-second forced expiratory volume (FEV_1_), and maximal mid-expiratory flow rate (MMF).

We performed skin prick tests using a Quick-Test Applicators (Quanti-Test system of Panatrex Co., California, USA) with 16 common aeroallergens (Dermatophagoides pteronyssinus, Dermatophagoides farinae, house dust, cockroach, cat hair, dog dander, Alternaria, Aspergillus mix, Albicans, Cadosporium, Penicillin mix, Eucalyptus, Ragweed, Bermuda, cotton, and feather) and aqueous extracts of teas prepared by standard techniques [17]. Positive (histamine) and negative (saline) controls were also applied. Skin reactions were read 20 min afterwards, and a test was considered positive if the diameter of the wheel was at least 3 mm greater than the control.

From each participant, we collected a venous blood sample for the radioallergosorbent test (RAST; Pharmacia CAP system, Uppsala, Sweden) for specific immunoglobulin (IgE) antibodies against *Camellia sinensis*. We performed this assay according to the manufacturer's instructions and calibrated using a standard preparation. A test was considered as positive if the result was greater than 0.35 ku/l on the scale of 1 to 6.

### Assessments of pulmonary function, sensitization status, and tea dust exposure during work

The during-work assessments were performed on the tea workers from September to October during the autumn tea production term in the same year as the baseline assessments. Pulmonary function tests were conducted at work about 5 days after the initiation of manufacturing. The instrument, method, and technician were the same as in the baseline assessment.

Four factories were randomly selected for tea dust measurements, and personal samples were collected during the whole work shift for three processes--withering, panning, and ball-rolling. We performed active sampling using GilAir ™ pumps (flow rate 2 L/min; Gilian Instrument Co., USA) with IOM samplers (SKC Inc., Eighty Four, PA, USA) and poly-vinyl chloride (PVC) filters (GLA 5000™, 25 mm diameter, 5 μm pore size) for inhalable dusts, and GilAir ™ pumps (flow rate 1.7 L/min; Gilian Instrument Co., USA) with Nylon cyclones samplers (MAS, Pittsburg, PA, USA), 37-mm filter cassette (SKC Inc., Eighty Four, PA, USA) and PVC filters (37 mm diameter and 5.0 μm pore size) for respirable dusts [18].

To assess changes in specific IgE against tea extracts, we collected venous blood samples again at work 5 days after the initiation of manufacturing. The methods were as the same as in the baseline assessment.

### Statistical analysis

In addition to respiratory symptoms and pulmonary function test results, we compared demographic characteristics including age, race, height, weight, and education level, as well as smoking and tea-drinking habits between the tea and office workers and among different subgroups of tea workers. Differences between the tea and office workers were evaluated using two-sample *t *test, chi-square test, or Fisher's exact test, and differences among different subgroups of tea workers were evaluated using ANOVA, chi-square test, or Fisher's exact test. To evaluate the effect of each variable, we used multiple linear regressions. Furthermore, we evaluated the changes in pulmonary function during work shift in each subgroup of tea workers using paired *t *test and between two subgroups using two-sample *t *test. We performed all the statistical analyses using SPSS 11.0 for Windows (SPSS Inc., Chicago, USA).

## Results

Of the 135 candidates, 93 tea workers and 33 office workers participated in this study, yielding a participation rate of 93.3%. However, two tea workers and one office worker were excluded due to existing diseases. The final study population of 91 tea workers and 32 office workers (the comparison group) had similar age and smoking habits, indicating successful matching (Table 1). The two groups also had similar height, weight, and distribution of race, but tea workers drank tea more frequently. The comparison group had higher education levels because of the requirements for obtaining the jobs.

**Table 1:** Demographic characteristics and baseline pulmonary function in the tea worker and comparison groups

Characteristics	Tea Workers (N = 91)	Comparison (N = 32)	*p*
Age (year) (mean ± SD)	40.6 ± 10.4	41.1 ± 9.1	0.81
Height (cm) (mean ± SD)	167.7 ± 6.3	169.5 ± 5.1	0.15
Weight (kg) (mean ± SD)	67.2 ± 8.1	69.6 ± 8.2	0.14
Education level*			< 0.01
Elementary	18 (19.8%)	0 (0.0%)	
Junior high	27 (29.7%)	0 (0.0%)	
Senior high	45 (49.5%)	26 (81.3%)	
University	1 (1.1%)	6 (18.7%)	
Race			0.80
Han	83 (91.2%)	30 (93.8%)	
Aboriginal	7 (7.7%)	2 (6.2%)	
Hakka	1 (1.1%)	0 (0.0%)	
Smoking			0.86
Never	31 (34.1%)	10 (31.2%)	
Ex-smoker	16 (17.6%)	7 (21.9%)	
Current	44 (48.3%)	15 (46.9%)	
Tea-drinking*			< 0.01
Never	3 (3.3%)	8 (25.0%)	
Occasional	22 (24.2%)	11 (34.4%)	
Habitual	66 (72.5%)	13 (40.6%)	
Baseline pulmonary function^†^			
FVC (L) (mean ± SD)	4.05 ± 0.68	4.15 ± 0.58	0.41
FVC (%pred^‡^) (mean ± SD)	89.10 ± 10.38	89.42 ± 7.27	0.85
FEV1 (L) (mean ± SD)	3.66 ± 0.64	3.77 ± 0.59	0.39
FEV1 (%pred) (mean ± SD)	97.47 ± 12.63	98.30 ± 9.47	0.70
FEV1/FVC (mean ± SD)	90.47 ± 4.81	90.60 ± 4.67	0.90
MMF (L/S) (mean ± SD)	4.93 ± 1.29	4.99 ± 1.12	0.79
MMF (%pred) (mean ± SD)	123.78 ± 30.37	123.44 ± 23.73	0.95

*p value < 0.05 for two-sample *t *test, chi-square test, or Fisher's exact test

^†^FVC: forced vital capacity, FEV1: 1-second forced expiratory volume, MMF: maximal mid-expiratory flow rate; ^‡^pred = predicted value

According to the task, we divided the tea workersinto four subgroups: withering (W), withering and panning (WP), ball-rolling (B), and withering and panning plus ball-rolling (WPB). The subgroups were similar in terms of height, weight, education level, race, and smoking and tea-drinking habits, but the B subgroup were younger than the others due to the requirement of more extensive labor (Table 2). We found that ball-rolling had the highest inhalable dust level in most of the factories and panning had the highest respirable dust level in all the factories (Table 3). Withering had the lowest levels of both dusts. Ball-rolling also had the highest coarse fraction (defined as inhalable dusts minus respirable dusts), which representsexposures from nose to tracheobronchial tract.

**Table 2:** Demographic and baseline pulmonary function among tea workers engaged in different processes

	Withering (N = 13)	Withering+Panning (N = 35)	Withering+Panning+Ball-Rolling (N = 19)	Ball-Rolling (N = 22)	p*
Age (year)* (mean ± SD)	43.7 ± 13.5	43.3 ± 9.6	38.8 ± 9.9	35.7 ± 7.9	0.02
Height (cm) (mean ± SD)	166.8 ± 5.9	166.1 ± 6.5	168.2 ± 6.4	169.9 ± 5.9	0.15
Weight (kg) (mean ± SD)	68.6 ± 11.1	65.2 ± 7.2	68.3 ± 7.9	68.1 ± 7.5	0.39
Education level					0.26
Elementary	4 (30.8%)	8 (22.9%)	4 (21.1%)	1 (4.5%)	
Junior high	2 (15.4%)	11 (31.4%)	4 (21.1%)	10 (45.5%)	
Senior high	7 (53.8%)	16 (45.7%)	10 (52.6%)	11 (50.0%)	
University	0 (0.0%)	0 (0.0%)	1 (5.2%)	0 (0.0%)	
Race					0.59
Han	11 (84.6%)	31 (88.6%)	19 (100.0%)	20 (90.9%)	
Aboriginal	2 (15.4%)	3 (8.6%)	0 (0.0%)	2 (9.1%)	
Hakka	0 (0.0%)	1 (2.8%)	0 (0.0%)	0 (0.0%)	
Smoking					0.08
Never	8 (61.5%)	10 (28.6%)	6 (31.6%)	6 (27.3%)	
Ex-smoker	2 (15.4%)	10 (28.6%)	3 (15.8%)	1 (4.5%)	
Current	3 (23.1%)	15 (42.8%)	10 (52.6%)	15 (68.2%)	
Tea-drinking					0.93
Never	0 (0.0%)	1 (2.9%)	1 (5.3%)	1 (4.5%)	
Occasional	3 (23.1%)	7 (20.0%)	5 (26.3%)	7 (31.8%)	
Habitual	10 (76.9%)	27 (77.1%)	13 (68.4%)	14 (63.7%)	
Baseline pulmonary function^†^					
FVC (L) (mean ± SD)	3.81 ± 0.57	3.89 ± 0.61	4.23 ± 0.75	4.24 ± 0.69	0.08
FVC (%pred^‡^) (mean ± SD)	87.72 ± 8.10	88.59 ± 10.57	90.91 ± 10.96	88.59 ± 11.12	0.82
FEV1 (L) (mean ± SD)	3.49 ± 0.47	3.57 ± 0.57	3.84 ± 0.75	3.69 ± 0.65	0.34
FEV1(%pred) (mean ± SD)	98.25 ± 9.80	98.38 ± 12.64	99.53 ± 13.51	92.63 ± 12.34	0.26
FEV1/FVC (mean ± SD)	91.98 ± 2.05	91.74 ± 4.60	90.79 ± 4.57	86.94 ± 4.98	< 0.01
MMF (L/S) (mean ± SD)	4.97 ± 0.79	4.85 ± 1.19	5.28 ± 1.44	4.63 ± 1.47	0.44
MMF (%pred) (mean ± SD)	133.42 ± 20.0	125.79 ± 3 0.9	128.21 ± 30.5	108.32 ± 30.7	0.05

*p value for ANOVA, chi-square test, Fisher's exact test

^†^*FVC *forced vital capacity, *FEV1 *1-second forced expiratory volume, *MMF *maximal mid-expiratory flow rate, ^‡^pred = predicted value

**Table 3:** Environmental tea dust measurements of four tea manufacturing factories

Factory	Inhalable Dusts (mg/m^3^)			Respirable Dusts (mg/m^3^)			Coarse Fraction (mg/m^3^)		
	
	Withering	Panning	Ball-Rolling	Withering	Panning	Ball-Rolling	Withering	Panning	Ball-Rolling
A	0.81	1.25	2.51	0.19	0.74	0.29	0.62	0.51	2.22
B	0.93	3.24	2.32	ND*	0.74	0.27	0.93	2.5	2.05
C	1.19	1.79	5.61	0.41	0.60	0.55	0.78	1.19	5.06
D	1.13	1.83	2.64	0.27	1.04	0.25	0.86	0.79	2.39

Mean ± SD	1.01 ± 0.18	2.03 ± 0.85	3.27 ± 1.57	0.21 ± 0.17	0.78 ± 0.19	0.34 ± 0.14	0.80 ± 0.13	1.25 ± 0.88	2.93 ± 1.43

*ND non-detectable

The prevalence of atopy to at least one of the common aeroallergens was similar between the two groups (27.5% vs. 28.1%, *p *> 0.05). The most common sensitization was that to house dusts (13.8%), followed by Dermatophagoides farinae (8.9%), cat hair (8.9%), and cockroach (6.5%). Only one participant, a tea worker in the W subgroup, was sensitized to tea as demonstrated by skin prick tests as well as baseline and during-work RASTs (baseline: +, during-work: 2+).

**Figure 2:**
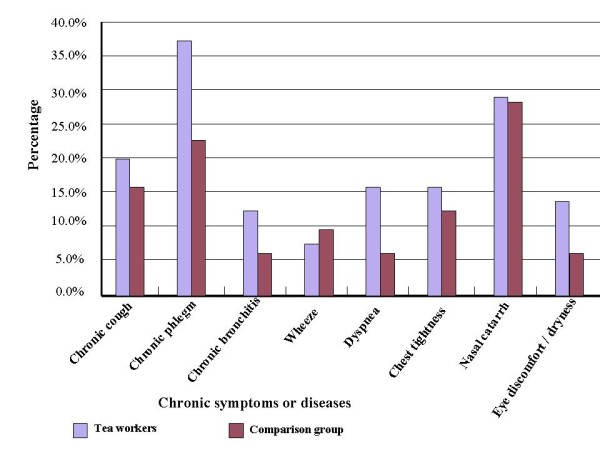
**The prevalence of chronic respiratory symptoms in tea workers and the comparison group**.

**Figure 3:**
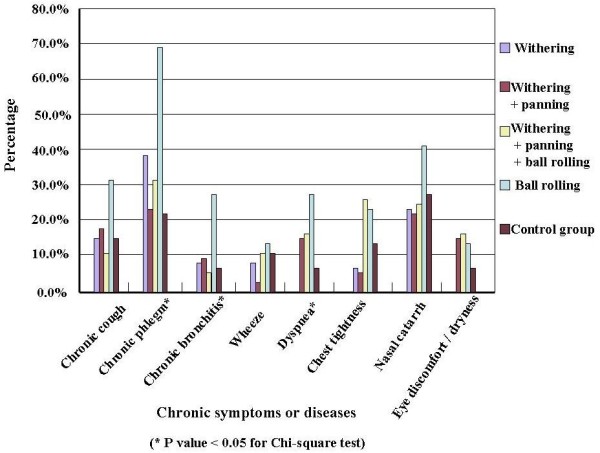
**The prevalence of chronic respiratory symptoms in tea workers classified by work schedule**.

**Figure 4:**
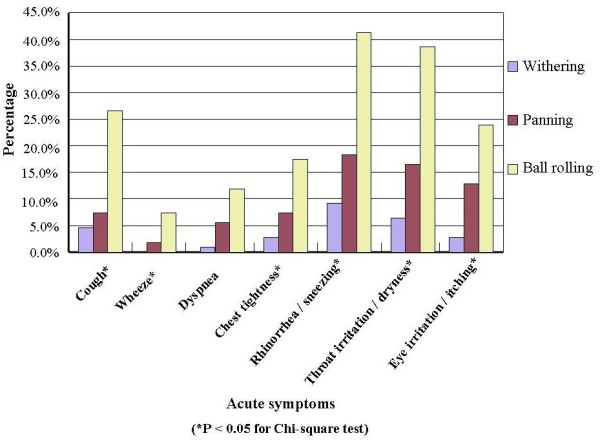
**The prevalence of work-related respiratory symptoms in different manufacturing processes of tea**.

In comparison with office workers, tea workers had higher prevalence of most chronic symptoms (Figure 2). The four subgroups of tea workers had different baseline prevalence of chronic phlegm, chronic bronchitis, and dyspnea, even after adjusting for age, and the B subgroup had the highest prevalence of most chronic symptoms (Figure 3). In tea workers, the most common work-related symptoms were rhinorrhea/sneezing, throat irritation, and cough, and workers engaged in different processes had different prevalence of all the work-related symptoms except for dyspnea. Among the processes, ball-rolling was associated with the highest prevalence of all work-related symptoms (Figure 4).

Tea workers and the comparison group had similar baseline pulmonary function tests (Table 1). In tea workers, the B subgroup had the lowest FEV_1_/FVC and MMF expressed as% of the predicted value--MMF (%pred) (Table 2). In comparison with office workers, the B subgroup still had lower FEV_1_/FVC (86.94% vs. 90.60%) and MMF (%pred) (108.32% vs. 123.44%). Five participants, all were tea workers, had abnormal baseline pulmonary function defined as having an FVC or FEV_1 _smaller than 80% of the predicted value or an FEV_1_/FVC smaller than 75%. All of them had been tea workers for more than 10 years and were engaged in ball-rolling or mixed processes, and most of them had multiple chronic symptoms. After adjusting for age, height, weight, work duration, smoking and tea-drinking, the work task (as defined by the subgroups) was an independent predictor of both reduced FEV_1_/FVC and MMF (Table 4).

**Table 4:** Multiple regression analyses of pulmonary function data^†^

Variable	FVC (L)		FEV 1(L)		FEV1/FVC (%)		MMF (L/S)	
	
	Beta [SE^‡^]	*p*	Bet [SE]	*p*	Beta [SE]	*p*	Beta [SE]	*p*
Model 1: by work tasks								

Task^#^								
WP vs. W	0.122 [0.166]	0.47	0.113 [0.165]	0.50	-0.148 [1.476]	0.92	-0.202 [0.392]	0.61
WPB vs. W	0.267 [0.182]	0.15	0.204 [0.180]	0.26	-1.341 [1.614]	0.41	-0.022 [0.429]	0.96
B vs. W	0.129 [0.185]	0.49	-0.084 [0.183]	0.65	-5.200 [1.640]	< 0.01	-0.845 [0.436]	0.05
Age (year)	-0.028 [0.006]	< 0.01	-0.029 [0.006]	< 0.01	-0.099 [0.053]	0.07	-0.061 [0.014]	< 0.01
Height (cm)	0.046 [0.010]	< 0.01	0.033 [0.010]	< 0.01	-0.216 [0.087]	0.01	-0.013 [0.023]	0.57
Weight (Kg)	-0.001 [0.007]	0.94	0.003 [0.007]	0.71	0.065 [0.063]	0.30	0.012 [0.017]	0.37
Smoking								
Ex-smoker vs. Never	0.007 [0.163]	0.97	-0.013 [0.161]	0.94	-0.446 [1.442]	0.76	0.158 [0.383]	0.68
Current vs. Never	-0.111 [0.122]	0.36	-0.139 [0.120]	0.25	-1.007 [1.079]	0.35	-0.195 [0.287]	0.50
Tea-drinking								
Occasional vs. Never	0.170 [0.302]	0.57	0.208 [0.299]	0.49	1.257 [2.677]	0.64	-0.007 [0.711]	0.99
Habitual vs. Never	0.300 [0.294]	0.31	0.225 [0.291]	0.44	-1.091 [2.609]	0.68	-0.521 [0.693]	0.45
Annual work duration (month)	0.001 [0.003]	0.64	0.002 [0.003]	0.41	0.035 [0.027]	0.20	0.016 [0.007]	0.03
Model 2: by dust level								
Coarse fraction	0.033 [0.070]	0.64	-0.073 [0.070]	0.30	-2.612 [0.615]	< 0.01	-0.332 [0.166]	0.04
Respirable dust	-0.190 [0.466]	0.68	-0.265 [0.463]	0.57	-2.739 [4.069]	0.50	-0.612 [1.099]	0.58
Age (yea)	-0.028 [0.006]	< 0.01	-0.030 [0.006]	< 0.01	-0.104 [0.053]	0.05	-0.062 [0.014]	< 0.01
Height (cm)	0.045 [0.010]	< 0.01	0.032 [0.010]	< 0.01	-0.230 [0.087]	0.01	-0.016 [0.024]	0.49
Weight (Kg)	-0.001 [0.007]	0.88	0.002 [0.007]	0.76	0.067 [0.062]	0.29	0.014 [0.017]	0.42
Smoking								
Ex-smoker vs. Never	0.046 [0.162]	0.77	0.032 [0.161]	0.84	-0.241 [1.416]	0.87	0.163 [0.382]	0.67
Current vs. Never	-0.086 [0.120]	0.47	-0.112 [0.119]	0.35	-0.912 [1.046]	0.39	-0.208 [0.283]	0.46
Tea-drinking								
Occasional vs. Never	0.134 [0.302]	0.66	0.170 [0.301]	0.57	1.166 [2.643]	0.66	-0.026 [0.714]	0.97
Habitual vs. Never	0.270 [0.295]	0.36	0.198 [0.293]	0.50	-1.054 [2.574]	0.68	-0.520 [0.696]	0.46
Annual work duration (month)	0.002 [0.003]	0.57	0.003 [0.003]	0.34	0.038 [0.026]	0.15	0.016 [0.007]	0.03

^†^*FVC *forced vital capacity, *FEV1 *1-second forced expiratory volume, *MMF *maximal mid-expiratory flow rate; ^‡^*SE *standard error; ^#^*W *withering, *WP *Withering + Panning, *WPB *Withering + Panning + Ball-Rolling, *B *Ball-Rolling

According to the mean inhalable and respiratory dust levels and work task, we calculated every tea worker's dust exposure levels, and the level of coarse fraction was still an independent predictor of both reduced FEV_1_/FVC and MMF. Fifty-eight tea workers participated in the during-work assessments, including 39 withering workers and 19 ball-rolling workers. We observed declines in FVC, FEV1, and FEV1/FVC, but not MMF. (Table 5) Compared to withering workers, ball-rolling workers had greater changes in pulmonary function.

**Table 5:** Changes in pulmonary function^† ^during work

		All subgroups (N = 58)	Withering subgroup (N = 39)	Ball-rolling subgroup (N = 19)
FVC	%	-3.64	-3.33	-4.26
	p*	< 0.01	< 0.01	< 0.01
	p**		0.59	
FEV1	%	-4.71	-4.05	-6.05
	p*	< 0.01	< 0.01	< 0.01
	p**		0.28	
FEV_1_/FVC	%	-1.11	-0.74	-1.87
	p*	0.04	0.24	0.05
	p**		0.36	
MMF	%	0.50	0.08	1.38
	p*	0.34	0.42	0.62
	p**		0.84	

*p values for paired t tests in comparison with the baseline values

**p values for two-sample t tests between the withering group and the ball-rolling group

^†^*FVC *forced vital capacity, *FEV1 *1-second forced expiratory volume, *MMF *maximal mid-expiratory flow rate

## Discussion

The amount and size of dusts are two important factors of the respiratory effects of tea dusts. In a study on the sorting and grinding of dried fruits and teas, the total dusts ranged from 8.3 to 24.9 mg/m^3 ^with a mean of 16.8 mg/m^3^, and the respirable fraction ranged from 1.0 to 6.4 mg/m^3 ^with a mean of 3.2 mg/m^3 ^[11]. However, area sampling assesses the distribution of dusts in the environmental, which does not necessarily reflect the worker's true exposure. We used personal sampling and found that panning and withering had higher respirable fractions (0.78/2.03 and 0.21/1.01, respectively) than ball-rolling (0.34/3.27), although ball-rolling had the most inhalable dusts. These results indicate that panning and withering produce smaller tea dusts, and ball-rolling generates larger dusts. Inhalable dusts can reach the respiratory tract anywhere from the nose to alveoli and cause effects throughout the entire respiratory system, while respirable dusts, with smaller particle sizes, can enter the alveolar region of the lung and cause fibrosis or pneumoconiosis that may be represented by changes in FVC [19]. The coarse fraction represents exposures from the nasal cavity to tracheobronchial tract, and ball-rolling has the highest exposure level. This may be related to respiratory tract obstruction that can be represented by changes in FEV_1 _and MMF.

In our study, tea workers and the comparison group had similar prevalence and pattern of atopy to common aeroallergens, and a previous study on common tea packers observed the same [8]. We found only one case allergic to tea, which is also compatible to findings in that previous study [8], but some other studies on workers processing fruit and herbal teas reported higher prevalence [11,12]. This may be due to the differences in allergenicity between common tea and other types of teas. Because of the low prevalence of sensitization to tea, the respiratory effects of tea dust exposure are more likely to represent an irritation response, rather than an allergic reaction.

In our study, tea workers had higher prevalence of almost all chronic symptoms than the comparison group, and in further analyses, we found the B subgroup had higher prevalence of all symptoms than the other tea workers. Whereas dust levels in our study were lower, we observed prevalence of chronic and work-related symptoms similar to those reported by previous studies [8,10,11], except for chronic phlegm, which might be due to the high prevalence of smoking and low prevalence of respiratory protecting equipment use (less than 2%) in our study. In addition to higher tea dust levels, ball-rolling is more labor-intensive, which may increase the ventilation rate and dust intake and thus increase the symptoms [20]. This might be the reason why the B subgroup had higher prevalence of chronic and work-related symptoms.

Although tea workers and the comparison group had similar baseline pulmonary function tests, the B subgroup still had worse pulmonary functions than the other tea workers and the comparison group. This is compatible to the results of tea dust assessment and reported respiratory symptoms. The abnormal pulmonary function tests we observed were mainly obstructive changes, similar to those observed in previous studies [9,11,12]. However, among our cases, two had combined obstructive and restrictive impairment. Therefore, further studies should be conducted to evaluate if long-term high-level tea dust exposure can lead to restrictive changes. Among tea workers, significant declines during work were observed in FVC, FEV_1_, and FEV1/FVC, but not MMF. MMF is a sensitive parameter for early detection of smaller airways obstruction but has larger variations even using standard testing procedure [10,21], which may affect the results of comparisons. Withering was also associated with during-work declines in pulmonary function, which might be due to the fact that some workers had performed ball-rolling or panning before the tests.

The multiple regression analyses showed that age and height were independent predictors of pulmonary function, which is a well documented fact [1,21]. It should be noted that job task also appeared to be an independent predictor of FEV_1_/FVC. The coarse fraction was an independent predictor of both FEV_1_/FVC and MMF, which was compatible with the effects of dusts on the tracheobronchial tracts. Furthermore, the number of months worked each year had positive associations with all the parameters of pulmonary function and was an independent predictor of MMF. This might be attributable to the fact that healthier worker can work longer, a phenomenon known as "healthy worker effect". Whereas smoking is associated with declines in pulmonary function, it was not an independent predictor of pulmonary function in our study. The classification of smoking habits in our study was simple, which might lead to remarkable variations within each category as reflected by the relatively large standard errors of the regression coefficients. The healthy worker effect might also contribute to this observation. Further studies with larger numbers of participants that allow stratification of smoking habit in greater details are warranted to better evaluate the effect of smoking. In the analysis of the changes in pulmonary function during work, workers engaged in withering also had declines in FVC and FEV_1_, which might be due to the fact that some workers had performed panning or rolling before the test. MMF is an early parameter of small airway obstruction and usually has large variations [10,21], which might be the main reason why MMF was not a sensitive outcome in our study.

The current study showed that exposure to tea dusts in the early processes of tea manufacturing, especially ball-rolling, could lead to the development of respiratory symptoms and impairment of pulmonary function. Therefore, proper preventive or control measures should be adopted [19,21-23]. Due to need of dry environment and manufacturing equipment, local exhaust ventilation and the wetting method are not suitable for this industry, and therefore effective general exhaust ventilation is required. In addition, personal respiratory protection such as simple disposable dust masks should be applied. Workers may also benefit from health surveillance and educational programs. In fact, Taiwan mandates annual health examinations, including pulmonary function tests and chest X-ray, for workers with exposure to dusts. Although smoking did not appear to be a significant predictor for respiratory symptoms and pulmonary function in our study, its effects to lung by themselves and by enhancing the toxicities of other occupational hazards with interference of pulmonary defense mechanisms are well documented [23,24]. Therefore, anti-smoking campaigns are still desirable in the tea industry.

## Conclusion

There are few studies on the respiratory effects of different processes in the early-stage tea manufacturing. We find that tea workers engaged in early manufacturing processes of tea are at risk of developing respiratory symptoms and pulmonary function impairment, which might be related to dust exposures. Whereas further studies with longitudinal follow-up designs and larger samples are warranted to confirm our findings, our study results have called for attention to the exposure to dusts among workers engaged in early manufacturing processes of tea.

## Abbreviations

B: ball-rolling; FEV_1_: 1-second forced expiratory volume; FVC: forced vital capacity; MMF: maximal mid-expiratory flow rate; MMF (%pred): maximal mid-expiratory flow rate expressed as% of the predicted value; PFT: pulmonary function; PFT: pulmonary function test; PVC: poly-vinyl chloride; W: withering; WP: withering and panning; WPB: withering and panning plus ball-rolling

## Competing interests

The authors declare that they have no competing interests.

## Authors' contributions

TS Shieh participated in the design of the study, performed the statistical analysis, and drafted the manuscript. JJ Chung and CJ Wang participated in the design of the study. PJ Tsai participated in the collection of environmental exposure and the assessment of collected data. YC Kuo participated in the interpretation of pulmonary function and drafted the manuscript. HR Guo conceived of the study, participated in the design and coordination of the study, and helped to draft the manuscript. All authors read and approved the final manuscript.

## Pre-publication history

The pre-publication history for this paper can be accessed here:

http://www.biomedcentral.com/1471-2458/12/121/prepub
